# Mechanisms of offline motor learning at a microscale of seconds in large-scale crowdsourced data

**DOI:** 10.1038/s41539-020-0066-9

**Published:** 2020-06-04

**Authors:** Marlene Bönstrup, Iñaki Iturrate, Martin N. Hebart, Nitzan Censor, Leonardo G. Cohen

**Affiliations:** 1 Human Cortical Physiology and Neurorehabilitation Section, National Institute of Neurological Disorders and Stroke, Bethesda, MD 20814 USA; 2 Laboratory of Brain and Cognition, National Institute of Mental Health, Bethesda, MD 20814 USA; 3 School of Psychological Sciences and Sagol School of Neuroscience, Tel Aviv University, 69978 Tel Aviv, Israel

**Keywords:** Human behaviour, Consolidation

## Abstract

Performance improvements during early human motor skill learning are suggested to be driven by short periods of rest during practice, at the scale of seconds. To reveal the unknown mechanisms behind these “micro-offline” gains, we leveraged the sampling power offered by online crowdsourcing (cumulative *N* over all experiments = 951). First, we replicated the original in-lab findings, demonstrating generalizability to subjects learning the task in their daily living environment (*N* = 389). Second, we show that offline improvements during rest are equivalent when significantly shortening practice period duration, thus confirming that they are not a result of recovery from performance fatigue (*N* = 118). Third, retroactive interference immediately after each practice period reduced the learning rate relative to interference after passage of time (*N* = 373), indicating stabilization of the motor memory at a microscale of several seconds. Finally, we show that random termination of practice periods did not impact offline gains, ruling out a contribution of predictive motor slowing (*N* = 71). Altogether, these results demonstrate that micro-offline gains indicate rapid, within-seconds consolidation accounting for early skill learning.

## Introduction

Early learning of a new motor skill unfolds fast over the course of a single training session^1^. While performance improvements are typically linked to practice itself, recently it was shown that in fact, a substantial proportion of early skill acquisition happens during short rest periods in the range of several seconds that are interspersed with practice^2^. These micro-offline improvements closely track the early online learning curve before a performance plateau is reached, and account for total early learning. Micro-offline improvements during early skill learning may thus constitute a rapid form of consolidation, extending the concept of consolidation to a time-scale in the order of seconds, rather than hours or days, as has been a widely held assumption^3^.

Here, we directly tested this idea using a hallmark of consolidation referred to as stabilization^4^. Stabilization can be tested as the resistance of a memory trace to retroactive interference via a competing task^5^ or disruptive noninvasive brain stimulation^6^. Stabilization has been demonstrated to evolve hours after a full training session^3,7^. In the context of early skill learning, it is unknown, whether these short periods of rest, interspersed between practice bouts in an initial training session, allow for stabilization of the memory trace. Stabilization developing seconds after practice would strongly support the existence of consolidation acting at such a short temporal scale.

Alternatively, micro-offline gains may also be an indirect effect of performance fatigue with continuous practice^2,8^ that expresses as performance decrements, more specifically motor slowing. Such motor slowing is commonly seen during prolonged (>10 s) motor execution at maximum speed and, in its earliest appearance, is likely linked to a central disbalance between excitation and inhibition in motor cortical regions^9–12^. Recovery from motor slowing would lead to measurable performance improvements over subsequent rest periods, unassociated to within-rest learning^13–15^. Contrary to these observations, micro-offline gains documented during rest periods of early learning emerge in a fundamentally different context: they occur well before maximum speed is reached and are largest in trials that do not show within-practice performance decrements, i.e., in the absence of overt motor slowing. However, micro-offline improvements may arise from a recovery of potentially latent motor slowing during each preceding practice period, masked and counterweighted by micro-online improvements.

The aim was to disambiguate the presence of rapid consolidation mechanisms in early motor skill learning. We used the high sampling power of crowdsourcing and achieved large sample sizes in each experiment for replicable results^16,17^. Crowdsourcing has become a widely used, well-tested and versatile tool in social and cognitive sciences to investigate personality, decision making, visual perception and recognition, and memory formation^18^. It is used in replication studies^19^ and has recently also been adapted for motor learning research^20^.

We first tested the feasibility of studying human sequence learning dynamics in the web-based environment. This experiment also allowed to test the replicability of micro-offline gains during short periods of rest following each practice period in early human motor skill learning. Then, in a second experiment, we applied retroactive interference immediately after practice to test if short periods of rest, in the range of seconds, allow for stabilization of the memory trace to occur. In a third and fourth experiment, we experimentally manipulated the motor slowing effect to disambiguate the presence of consolidation mechanisms acting at the level of seconds.

## Results

### Early learning of a new skill occurs largely offline—replication in a large crowdsourced sample

**Fig. 1:**
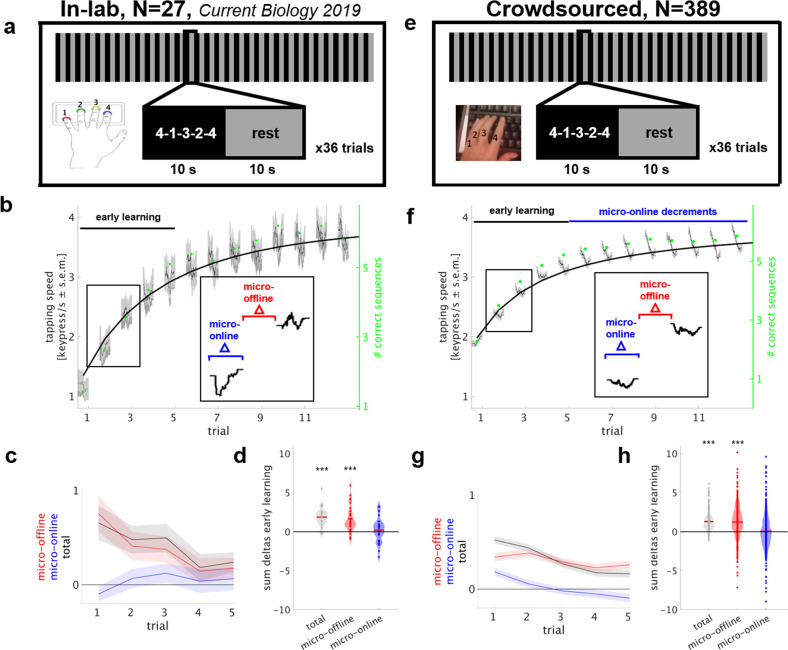
Experiment 1: early learning of a new skill occurs largely offline. Replication in a crowdsourced sample of 389 participants. **a**–**d** in-lab experiment (*N* = 27, 17 female, mean ± s.e.m. age 26.3 ± 0.83), **e**–**h** crowdsourced experiment (224 female, 39.6 ± 0.56) **a**, **e** Task: participants learned a motor skill task^2,22,23^ over 36 trials (inset shows a single trial) consisting of alternating practice and rest periods of 10 s duration for a total of 12 min. **b**, **f** Skill was measured as the average inter-tap interval within correct sequences (tapping speed measured in keypresses/s)^2,24^ and is shown over the first 11 trials for the in-lab (**b**) and crowdsourced (**f**) group (see Supplementary Fig. 1a, c for all 36 trials). Micro-online changes were calculated as the difference in tapping speed (keypresses/s) of the first and last correct sequence within a practice period (blue in inset) and micro-offline changes as the difference between the last correct sequence within a practice period compared with the first of the next practice period (red in inset)^2^. The average number of correct sequences per trial is shown as green dots. **c**, **g** Trial-wise early learning (trials 1–5) composed of micro-offline (red), micro-online (blue), and total (black) performance changes (mean + s.e.m.). Note the presence of large micro-offline gains and total early learning in the initial trials in the absence of micro-online performance decrements. **d**, **h** Data points in the violin plot depict the sum of changes in performance over early learning trials in each participant, the red line denotes the mean (***P < 0.001).

As a first step, we implemented an exact replication of the sequential finger tapping task^2,21^ in the web-based environment. This replication experiment serves two purposes: (1) to evaluate crowdsourcing as a tool for the study of dynamics of human motor learning behavior and (2) to replicate and extend to a larger and more representative sample our previous finding of micro-offline learning, demonstrating that a substantial portion of early skill learning is accounted for by performance improvements during short periods of rest. The motor learning task is well-characterized and widely used in the study of procedural motor memory formation^21^. It is comprised of a series of sequential keypresses that are executed repeatedly^22,23^. Overall 389 participants, recruited via the Amazon Mechanical Turk platform, trained over 36 trials, each consisting of 10 s practice and 10 s rest, for a total of 12 min (Fig. 1a, e and Table 1). In each practice period, participants were asked to repetitively tap a 5-item sequence indicated on the screen as fast and accurately as possible using their left, nondominant hand on the number keys on the left side of their own computer keyboard. Instructions were delivered via short and comprehensible statements alongside illustrations before the beginning of the task. Performance was measured as the tapping speed (keypresses/s) for correctly performed sequences^2,24^, which allows quantification of microscale, within-trial performance changes. And, as the number of correct sequences per trial, the classically used skill measure in this task^22,25,26^. Both measures were highly correlated, however less in the crowdsourced group as compared with the in-lab group (Pearson’s linear correlation coefficient over 36 trials in-lab group: *ρ* = 0.8 ± 0.03 mean ± s.e.m.; *N* = 27, 100% *P* < 0.05 and crowdsourced group: *ρ* = 0.73 ± 0.01; *N* = 389, 96% *P* < 0.05) and in the following, only the tapping speed is reported.

**Table 1:** Overview of experimental designs and participants.

Experiment	Experimental groups	trials *x* trial duration = total duration	Rest period duration	Practice period duration	Participants completed the task	Participants adherent to instruction	Participants included in analysis
1	1	36 × 20 s = 12 m	10 s	10 s	499	389	389, 212
2	3	12 × 40 s = 8 m			523	373	373
	Early interference		20 s	10 s seq A, 10 s seq B	178	118	118
	Late interference		20 s	10 s seq A, 10 s seq B	176	126	126
	No interference		30 s	10 s seq A	169	129	129
3	1	48 × 110 s = 12 m	10 s	5 s	438	249	118
4	1	41 × 15–20 s = 12 m	10 s	Jittered 5–10 s	249	170	71

In Experiment 3 and 4 (and the respective reference group from Experiment 1), we only included participants who showed a minimum baseline performance level of at least two completed sequences per trial.

Just as the in-lab motor learning dynamics (Fig. 1b), the performance of the crowdsourced, online acquired motor learning data (Fig. 1f) showed rapid performance improvements within the first few minutes of practice that constitute the early learning phase. Because most performance improvements occurred in those early trials and performance decrements during later trials confound the interpretation of performance improvements during subsequent rests, we focused our analyses of microscale learning dynamics to the early learning phase. Early learning in both groups was defined as the trials before which practice period performance significantly decremented in either group, which developed by trial 6 in the crowdsourced group (trial-by-trial two-tailed one-sample nonparametric permutation test, *P* < 0.05). Thus, early learning was defined as trials 1–5. At a high temporal resolution, strong performance increments between practice periods were evident (Fig. 1b, f). The contribution of practice and rest periods to total early learning was assessed as follows and congruent to previous work: micro-online learning was defined as the difference in tapping speed (keypresses/s) between the beginning and end of each practice period. Micro-offline learning was defined as the difference in tapping speed between the end of each practice period and the beginning of the next one (Fig. 1b, f, “Methods”)^2^. Total early learning was calculated as the sum of single-trial performance changes from trial 1–5. We found that micro-offline gains closely tracked trial-by-trial total early learning (Fig. 1c, g), which amounted to 1.31 ± 0.06 keypresses/s in the crowdsourced group and to 1.87 ± 0.23 keypresses/s in the in-lab group (mean ± s.e.m., two-tailed one-sample nonparametric permutation test, *P* < 0.001, group difference: two-tailed two-sample nonparametric permutation test, *P* < 0.01). This early learning was accounted for by performance improvements during rest periods rather than during practice periods. On average, micro-online changes were nil in both groups (crowdsourced group: 0.07 ± 0.12 keypresses/s, *P* = 0.72; in-lab group: 0.19 ± 0.30 keypresses/s, *P* = 0.48, group difference: *P* = 0.54) whereas micro-offline gains were substantial (crowdsourced group: 1.24 ± 0.12 keypresses/s; *P* < 0.001, Fig. 1h; in-lab group: 1.68 ± 0.32 keypresses/s; *P* < 0.001, group difference: *P* = 0.38, Fig. 1e). In addition to testing for differences between groups, we confirmed that microscale learning between groups was statistically equivalent (two one-sided test for equivalence, TOST). From trial 6 on, as tapping speed increased towards a plateau, within-practice performance started to decrement in both groups (Fig. 1, Supplementary Fig. 1).

Total session learning over all 36 trials amounted to 1.93 ± 0.05 keypresses/s in the crowdsourced group and to 2.73 ± 0.22 keypresses/s in the in-lab group (difference between trial 1 and 36, mean ± s.e.m., *P* < 0.001). The difference in total session learning and total early learning likely arose from difference in performance at trial 1, with the crowdsourced group showing higher starting performance than the in-lab group (1.17 ± 0.19 vs. 1.93 ± 0.06 keypresses/s, *P* < 0.001). This may be secondary to the specific subset of the worker population sampled at this given point in time^27^, or a potentially higher exposure to online and typing tasks in the worker population compared with participants in the lab. However, the ceiling level (trial 11–36 performance) was similar in both groups (3.67 ± 0.27 vs. 3.80 ± 0.05 keypresses/s, *P* = 0.63).

Although there were differences between the groups, the overall similarity between the groups with respect to the shape of the learning curve and microdynamics of motor learning, representing the variables of interest, replicated previously reported results. This demonstrates that the crowdsourcing marketplace is useful to study human motor learning dynamics at a large scale.

Having established a powerful tool for the fast collection of replicable human motor learning behavioral data, we went on to investigate the existence of consolidation mechanisms acting at the level of seconds.

### Stabilization of motor skill during short periods of rest

**Fig. 2:**
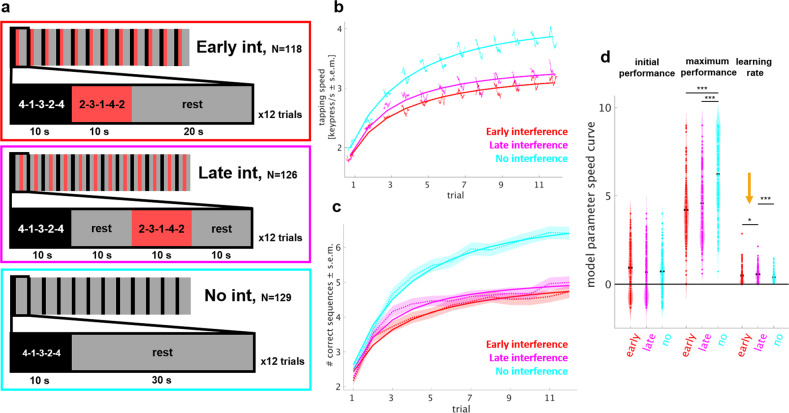
Experiment 2: stabilization of motor skill during short periods of rest. **a** Task: learning of the target sequence was interfered by learning of another sequence either immediately (early interference, *N* = 118, first row), or 10 s after (late interference, *N* = 126, second row) each practice period. To avoid proactive interference of late interference on the following trial, a rest period of 10 s was introduced and the rest period in the early interference group matched to 20 s. In a control group, no interference was given, and each practice period was followed by 30 s of rest (*N* = 129). Training consisted of 12 trials amounting to 8 min. **b** Skill was measured as the average inter-tap interval within correct sequences (tapping speed measured in keypresses/s)^2,24^ and **c** as the number of correct sequences^2,5,22,25,26^. The performance curve of each group (red: early interference, magenta: late interference, cyan: no interference; mean + s.e.m.) is overlaid with the average of modeled performance curves. Note the shallower rise of the performance curve in the early as opposed to late interference group. **d** Model parameters (initial performance, maximum performance, and learning rate) of the learning curve for each experimental group, the line denotes the mean. Modeling of the number of correct sequences revealed a significant group difference in the learning rate, with early interference showing a shallower curve (*P* < 0.05, orange arrow). *P < 0.05, ***P < 0.001.

The previous results document one hallmark of consolidation: performance enhancements^4^. However, consolidation may also express as stabilization of the newly acquired memory trace over periods of rest. For example, interference after a complete training session on a new motor skill has been shown to disrupt consolidation if applied shortly after training, but not if applied after the passage of time^5,7,28^. Transferring this approach to the microscale of seconds, stabilization of a memory trace during periods of rest could be probed via interference with the memory trace at different timepoints during a given rest period. In a second experiment, we studied the effects of interfering on microscale learning. Subjects learned the same sequence as in Experiment 1. To probe the effects of interference on memory stabilization, subjects repeatedly practiced a second sequence of matched complexity (2-3-1-4-2)^5^, immediately after each practice period (“early interference”, *N* = 118) or after the passage of a 10 s rest (“late interference”, *N* = 126). To avoid proactive interference of the interfering sequence on the next practice period, we allowed another 10 s of rest to pass before the start of the new trial (Fig. 2a). A control group with “no interference” (*N* = 129) rested for 30 s to match the inter-practice period intervals. All groups trained on 12 trials for a total of 8 min (Table 1). The number of trials was reduced to minimize the duration of the task while still capturing the early dynamics of motor learning, when most performance improvements occur. Both groups of Experiment 1 showed that 95% of total session learning over all 36 trials was reached by trial 12 (assessed by modeling of the group average performance curve, see “Methods”). Data for each experimental group were collected simultaneously and tasks were randomly assigned to participants (see “Methods”).

Performance on the target sequence was quantified as tapping speed (keypresses/s) for correctly performed sequences^24^ (Fig. 2b) and number of correct sequences per trial^21^ (Fig. 2c). As expected, the “no interference” group showed a higher total session learning over all 12 trials as opposed to both interference groups (early interference: 1.25 ± 0.08 keypresses/s, 2.61 ± 0.08 correct seq/trial; late interference: 1.46 ± 0.09 keypresses/s, 2.77 ± 0.09 correct seq/trial; no interference: 1.78 ± 0.09 keypresses/s, 3.91 ± 0.09 correct seq/trial; mean ± s.e.m., group-wise comparison between “no interference” and each of the interference groups *P* < 0.001). See Supplementary Fig. 2 for the performance curves of the interfering sequences.

Learning curves based on tapping speed (Fig. 2b) and number of correct sequences (Fig. 2c) showed a difference between the early and late interference groups with a steeper rise in early trials (trial 1–5) and higher performance level during later trials (6–12). To quantify and compare learning parameters between groups, we devised a simple model that captures performance curves of each participant over all 12 trials. The model comprised three parameters representing the initial performance, maximum performance and learning rate (see Eq. 1, “Methods”, “Data Analysis” section). We then statistically compared the model parameters between the interference groups (Fig. 2d). The late interference group showed a higher learning rate compared with the early interference group (late: 0.26 ± 0.23, early: 2.15 ± 0.20, *P* = 0.04). The effect size of the group difference was small to medium (Cohen’s *d* 0.15)^29^. Similar differences with a stronger rise in the learning curve of a late interference groups vs. an early interference group were found in a smaller sample collected in the lab environment (Supplementary Fig. 3). The numerically smallest learning rate was estimated for the no interference group, although this group showed highest maximum performance. This seemingly discrepant result likely arises from the formulation of the model (“Methods” section, “Modeling of performance curves” section) in which the parameter learning rate is relative to the parameter maximum performance. In this sense, the smaller learning rate indicates the longer time the no interference group took to reach the relatively high maximum performance. Regarding early learning dynamics (trials 1–5), we found no differences in microscale learning parameters (micro-online/offline) or total early learning between both interference groups. However, all groups showed significant performance improvements during rest periods (micro-offline, early interference: 0.77 ± 0.19, late interference: 0.80 ± 0.19, *P* < 0.05, no interference: 1.30 ± 0.17 keypresses/s; *P* < 0.001), whereas performance improvements during practice periods were undetectable. The no interference group showed significantly stronger total early learning compared with both interference groups (early interference: 0.98 ± 0.09; late interference: 1.06 ± 0.10; no interference: 1.43 ± 0.10 keypresses/s both *P* = 0.003).

These results document memory stabilization at the temporal microscale with more prominent stabilization in the first 10 s after each practice period, suggestive of a temporal gradient.

### Micro-offline gains are independent of motor slowing during practice

During early learning (trials 1–5), it is theoretically possible that motor slowing develops in each practice period but is counterweighted by learning-related performance improvements. If this were the case, micro-offline gains during the subsequent rest period, could be secondary to a dissipation of latent motor slowing. As motor slowing is a function of practice time over the duration of training^12^, such a contribution could be tested by evaluating how reducing the duration of practice periods affects micro-offline gains. We reasoned that if micro-offline learning remains high when practice period duration is shortened, motor slowing effects are unlikely to represent the relevant driver.

**Fig. 3:**
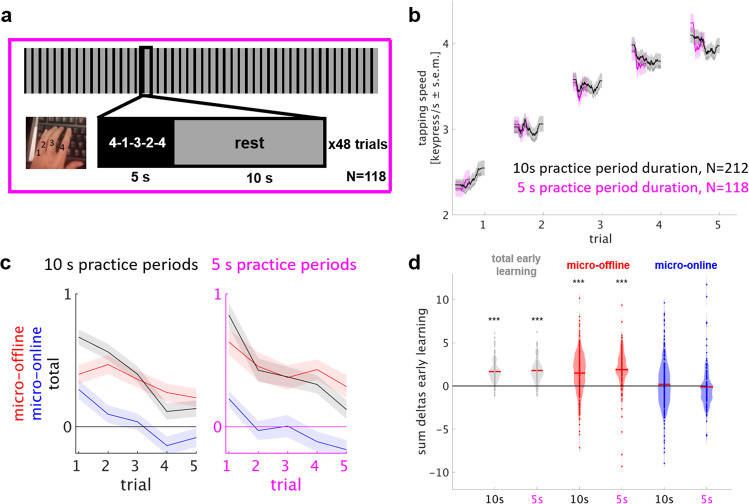
Experiment 3: training under reduced practice period duration shows comparable micro-offline gains. **a** Task: participants learned the motor skill task^2,22,23^ over 48 trials (inset shows a single trial) consisting of alternating 5 s practice and 10 s rest periods for a total of 12 min, matched to the original experiment duration (Fig. 1). **b** Skill was measured as the average inter-tap interval within correct sequences (tapping speed measured in keypresses/s)^2,24^. The group average performance curve is given in magenta (*N* = 118) and the group average of the 10 s practice period group (Experiment 1, Fig. 1) displayed for comparison in black (*N* = 212). **c** Trial-wise early learning. Each line depicts performance changes (micro-offline in red, micro-online in blue, total in black) per trial (mean + s.e.m.). Even under reduced practice period duration, total learning was closely accounted for by micro-offline gains (black and red lines) whereas micro-online performance changes fluctuate around 0 (blue line). Micro-offline learning remains high when halving practice period duration. **d** Data points in the violin plot depict the sum of changes in performance over early learning trials in each participant, the red line denotes the mean. In both groups (10 and 5 s), total early learning is accounted for by performance improvements during rest periods, but not during practice periods. ***P < 0.001. Note that participants with a high performance were selected in both groups due to required at least two correct sequences in each trial for calculation of microscale learning (“Methods”, “Data Analysis” section).

In Experiment 3, 181 participants trained the sequential finger tapping task in 48 trials alternating 5 s practice and 10 s rest periods (Fig. 3a), to match a total task duration of 12 min as in Experiment 1 (Table 1). Performance on the target sequence was quantified as tapping speed (keypresses/s) for correctly performed sequences. Early learning dynamics were compared with the original experimental group 1 that trained on alternating 10 s practice and 10 s rest periods. Both groups showed highly comparable performance during early learning trials (Fig. 3b). See Supplementary Fig. 4a for total session performance curves. In both groups, micro-offline gains closely tracked trial-by-trial total learning whereas micro-online changes remained relatively stable with performance stagnation, small decreases or increases (Fig. 3c). Total early learning was calculated as the sum of single-trial performance changes (trial 1–5) and amounted to 1.67 ± 0.08 keypresses/s in the 10 s practice period group as opposed to 1.79 ± 0.13 keypresses/s in the 5 s practice period group (mean ± s.e.m., *P* < 0.001, Fig. 3d). In both groups, total early learning was accounted for by performance improvements during rest periods rather than during practice periods. On average, micro-online changes were nil in both groups (10 s practice period group: 0.19 ± 0.20 keypresses/s, *P* = 0.48; 5 s practice period group: −0.10 ± 0.23 keypresses/s, *P* = 0.20) whereas micro-offline gains were substantial (10 s practice period group: 1.48 ± 0.17 keypresses/s, *P* < 0.001, Fig. 3d; 5 s practice period group: 1.89 ± 0.23 keypresses/s, *P* < 0.001). No significant differences in total early learning or microscale learning were evident (*P* > 0.05) and microscale learning between groups was statistically equivalent (TOST for equivalence). These results are inconsistent with a substantial contribution of motor slowing to micro-offline gains during early skill learning.

A closer look at later learning trials, in which within-practice performance decrements were evident (i.e., following trial 11, Supplementary Fig. 4a, b), suggested that motor slowing may develop faster within 5 s than 10 s practice periods. This finding could be explained if subjects preemptively slow down to stop as the end of each practice period approaches. In Experiment 4, we aimed to exclude preemptive slowing as a driver of micro-offline gains by having participants train the same task with practice periods of unpredictable duration.

**Fig. 4:**
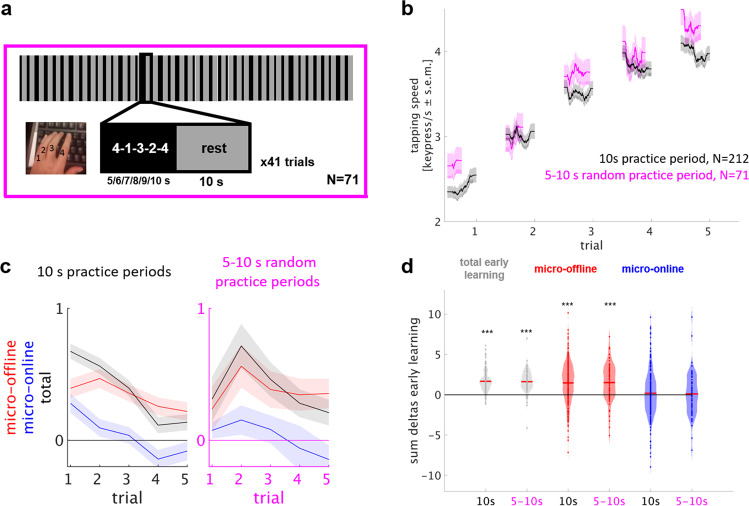
Experiment 4: training under unpredictable-practice period duration shows comparable micro-offline gains. Rhythmicity of practice-rest alterations may lead to preemptive slowing towards the end of each practice period that may contribute to micro-offline gains. Unpredictable-practice period durations (random 5, 6, 7, 8, 9, 10 s) prevent preemptive slowing. Task: participants learned the motor skill task^2,5,22^ over 41 trials (inset shows a single trial) consisting of alternating 5–10 s practice and 10 s rest periods for a total of 12 min, matched to the original experiment duration (Fig. 1). **b** Skill was measured as the average inter-tap interval within correct sequences (tapping speed measured in keypresses/s)^2,24^. The group average performance curve is given in magenta (*N* = 71) and the group average of the 10 s practice period group (Experiment 1, Fig. 1) displayed for comparison in black (*N* = 212). **d** Trial-wise early learning. Each line depicts performance changes (micro-offline in red, micro-online in blue, total in black) per trial (mean + s.e.m.). Even under unpredictable-practice period duration, total learning was closely accounted for by micro-offline gains (black and red lines) whereas micro-online performance changes fluctuate around 0 (blue line). **c** Data points in the violin plot depict the sum of changes in performance over early learning trials in each participant, the red line denotes the mean. In both groups (10 and 5 s), total early learning was accounted for by performance improvements during rest periods, but not during practice periods. ***P < 0.001, two-tailed, one-sample (within group) nonparametric permutation test for each learning partition. No across group comparison was significant. Note that participants with a high performance were selected in both groups due to required two correct sequences in each trial for calculation of microscale learning (“Methods”, “Data Analysis” section), thus trial 1 performance is comparably higher than in Experiment 1 and 2.

In Experiment 4, 71 participants trained the sequential finger tapping task in 41 trials of alternating 5, 6, 7, 8, 9, or 10 s (randomized order) practice and 10 s rest periods (Fig. 4a), to match a total task duration of 12 min as in Experiment 1 (Table 1). Early learning dynamics were compared with the original experimental group 1 who trained on alternating 10 s practice and 10 s rest periods. Both groups showed highly comparable performance during early learning trials (Fig. 4b). See Supplementary Fig. 4c for total session performance curves. In both groups, micro-offline gains closely tracked trial-by-trial total learning whereas micro-online changes remained relatively stable with stagnation, small decreases or increases (Fig. 4c). Total early learning was calculated as the sum of single-trial performance changes (trials 1–5) and amounted to 1.67 ± 0.08 keypresses/s in the 10 s practice period group as opposed to 1.63 ± 0.17 keypresses/s in the unpredictable-practice period group (mean ± s.e.m., *P* < 0.001, Fig. 4d). In both groups, total early learning was accounted for by performance improvements during rest periods rather than during practice periods. On average, micro-online changes were nil in both groups (10 s practice period group: 0.188 ± 0.02 keypresses/s, *P* = 0.50; unpredictable-practice period group: 0.11 ± 0.31 keypresses/s, *P* = 0.75) whereas micro-offline gains were substantial (10 s practice period group: 1.48 ± 0.17 keypresses/s; *P* < 0.001, Fig. 4d; unpredictable-practice period group: 1.52 ± 0.27 keypresses/s, *P* < 0.001). No significant differences in total early learning or microscale learning were evident (*P* > 0.05) and microscale learning between groups was statistically equivalent (TOST for equivalence). The groups differed with respect to trial 1 performance with the unpredictable-practice group showing higher starting performance than the 10 s practice group (2.68 ± 0.15 vs. 2.41 ± 0.07 keypresses/s, *P* < 0.05).

Together, the results of Experiments 3 and 4 are inconsistent with a dominant contribution of latent motor slowing to micro-offline gains and thus support consolidation as driving micro-offline gains.

## Discussion

Here, we first probed motor skill learning^21,30^ in the online crowdsourcing environment and then replicated a recent finding of performance improvements during early motor skill learning occurring predominantly during rest rather than during practice (Experiment 1). Two fundamentally different but not mutually exclusive mechanisms could theoretically contribute to micro-offline gains during early skill learning: rapid consolidation and latent motor slowing^8^. In a series of behavioral experiments, we showed that this effect cannot be explained by latent motor slowing resulting from within-practice performance fatigue (Experiments 3 and 4). We then found an indication of stabilization of the motor memory immediately after the end of each practice period (Experiment 2). Together, findings are consistent with consolidation evolving at the level of seconds and contributing to what is classically referred to as online learning^1^.

Motor skill training usually involves alternating periods of practice and rest. During initial training of a new motor skill, early performance improvements are substantial and develop over periods of rest that occur within a series of practice bouts within the same session. Our results in an in-lab (*N* = 27) and crowdsourced experiment (*N* = 389) coincided in showing that the sum of those micro-offline performance improvements substantially contributed to early learning, when most of the over all performance improvements manifested. Thus, micro-offline gains during early acquisition of a new motor skill make a sizable contribution to initial online learning^1^. This observation blurs the border between two stages of memory formation that are treated as distinct in neuropsychologic research. In fact, micro-offline gains speak to the contrary: behaviorally distinct stages of memory formation (online/offline learning, encoding/retrieval, and fast/slow) may not have isomorphic counterparts in brain plasticity but arise from a continuum of overlapping processes in brain physiology^31^. Short periods of rest offer a state of reduced interference compared with practice. A state of low interference, i.e., reduced external input and exposure to new experience, may be favorable for memories to consolidate synaptically^32^ and at the network level^33^. Previous work showed that at the network level, modulation of frontoparietal beta oscillatory activity during rest periods predicts micro-offline gains^2^, consistent with the involvement of this network, in encoding offline representations of movement kinematics^34^. It is possible that reactivation of previous practice-related activity^35,36^ or memory replay^37^ during rest intervals is embedded in low amplitude beta activity, an indicator of a state of sensorimotor engagement^38^. At the synaptic level, within-seconds micro-offline improvements in performance are likely the result of transient synaptic facilitation, mediated via modifications of the ionic microenvironment and briefly altered membrane conductance following increased firing activity^39^. Transient perturbations at the cellular (molecular, synaptic) level influence, and are integrated into, the formation of lasting, stable states at the at the circuit and network level^31^. Since memories form over several nested temporal scales, it remains to be determined in how far rapid and longer forms of consolidation are independent processes or serially linked. The in-lab experimental group was tested after 24 h and overnight offline learning did not correlate with micro-offline gains during early learning, suggesting different mechanisms at play^2^.

Memory consolidation refers to the process by which a temporary, labile memory is transformed into a more stable, long-lasting form^3^. Consolidation may not only express as performance enhancements over periods of rest (offline gains), a feature demonstrated in Experiment 1, but also as resistance to interference (stabilization^4^). Resistance to interference with the passage of time is a feature of consolidation of explicit memories^40^ and dynamic motor adaptation^7,28^. It has been tested by learning a competing task^5^, disrupting brain activity with noninvasive brain stimulation^6^ or by evaluating the effects of pharmacological inhibitors^41^ after a full learning session. If micro-offline gains constitute a form of ultrafast consolidation, stabilization of the memory trace may be experimentally tested at the seconds level. To evaluate stabilization during early skill learning, we adapted a behavioral retroactive interference protocol^5^ to the level of single trials. Early retroactive interference led to a moderately shallower learning curve compared with late retroactive interference (Fig. 2, an effect not driven by baseline differences between groups), suggesting that stabilization of the memory trace in the initial 10 s post practice rendered it more resistant to interference in the late interference group. This finding is reminiscent of the temporal gradient of memory stabilization reported in longer forms of consolidation^40^. We did not find, however, differences in microscale learning dynamics (micro-online, -offline contribution to total early learning, total early learning, total session learning) across groups. Our finding of relatively subtle differences in stabilization between the early and late interference groups is in line with the mixed results reported on retroactive interference effects in sequence learning as well as kinematic adaptation^5,42^. In dynamic motor adaptation learning, stabilization effects are more robustly revealed via retroactive interference^28^. This possibly reflects differences in neural systems^3,43^, neurophysiology and cellular circuit processing involved^3^. The combined declarative and procedural features of our task, particularly in early trials, when the short sequence is also processed declaratively, could have contributed to the relatively small magnitude of the effect^30^.

A fundamentally different mechanisms from consolidation that could theoretically express as micro-offline gains during early skill learning is recovery from latent motor slowing^8^. In contrast to strong micro-offline improvements during rest, micro-online changes during early learning (trial 1–5) were on average nil. Despite the absence of within-practice performance decrements in these early trials, latent motor slowing theoretically could contribute to micro-offline gains. In this scenario motor slowing is not strong enough to induce micro-online performance decrements but is enough to neutralize micro-online performance gains, resulting in performance stagnation over trials 1–5 (Fig. 1c, g). We addressed this possibility with the following reasoning: motor slowing is a function of practice time^12,14^, a reduction of practice period duration must therefore reduce the impact of possibly latent motor slowing on micro-offline gains. In Experiments 3 and 4, we found that micro-offline learning accounted for the totality of early learning even when practice period duration was substantially reduced by 50% and when practice period termination was unpredictable (Fig. 3c, d, 4c, d). Thus, latent motor slowing due to prolonged practice duration, or preemptive slowing due to the anticipation of the end of practice, are not relevant contributors to micro-offline gains during early skill learning.

At later learning stages (beyond trial 11), after a performance plateau was reached, within-practice performance decrements largely explained late learning micro-offline gains. Performance decrements that robustly express during continued and intense motor output, are an expression of motor fatiguability and in their earliest appearance, seem to be related to supraspinal mechanisms^44,45^. A shift in the excitation/inhibition balance with a decrease in inhibitory and increase in facilitatory cellular circuits at the motor cortex^9,10,12,44^ which normalizes to baseline levels within seconds after movement termination^12^.

The existence of learning under fatigue conditions has remained inconclusive so far. And Experiments 3 and 4 even challenge the common idea that “practice makes perfect” under no fatigue conditions. Total early learning in trials 1–5, before performance decrements indicate fatigue, was equivalent in groups practicing for 5, 6, 7, 8, 9, or 10 s, thus learning seemed to be independent of the amount of practice. Even short practice periods may trigger processes that lead to skill acquisition with a temporal delay. This finding is in line with previous reports showing that brief training reactivations may be as effective as longer practice periods to learn in both perceptual and motor domains^46^. In long practice intervals, lengthy practice may hide this temporal delay and so the learning is (mis)attributed to come from practice but offline processing is occurring latently during practice^8^. The amount of practice to trigger such offline processing may vary from task to task and interindividually but for the skill of sequential tapping, our results indicate that the amount of practice sufficient to trigger latent or overt offline stabilization, and thus learning, ceils at or even before 5 s of practice.

Apart from replicability of microlearning dynamics, our results demonstrate that crowdsourcing, a versatile tool in social and cognitive sciences^18–20^ can be used to study human motor skill learning. We developed a web-based version of a typical motor skill learning task^5,21,22^ keeping all aspects of the task identical except for the fact that participants used their own computer hardware and carried out the task in their chosen environment. The overall shape of the learning curves with a steep rise in early trials (1–5), transition to a plateau phase starting by trial 6–11, robust performance decrements during late learning trials and the performance ceiling level were highly replicable across crowdsourced and in-lab experiments. Importantly, there were no statistical differences of micro-online/-offline learning dynamics during early learning between crowdsourced and in-lab experimental groups. Differences between the two experimental groups included performance in the first trial, with the Experiment 1 crowdsourced group showing higher performance relative to the in-lab group. Consecutively, the in-lab group had higher total session learning and total early learning. As the performance in the Experiment 1 crowdsourced group is comparable with the groups in Experiment 2 as well as other in-lab experimental groups (1.71 ± 0.15 and 1.86 ± 0.24, Supplementary Fig. 5), we conclude, that the in-lab group engaged participants with lower starting performance than expected in the general population. These results highlight the contribution of crowdsourcing platforms making large-scale samples accessible for the study of properly powered and reproducible motor learning behavior. Ethical issues arising from the increasing use of crowdsourcing for human research should be part of the public discourse as personal interaction is minimal and the regulatory framework is harder to apply to internet research platforms. The monetary compensation should at least match the minimum wage, workers should be clearly informed about voluntary participation and ability to withdraw at any moment. They should be informed that their anonymity is limited by their worker ID and IP address being known to the researcher and, in case of contacting the researcher, all personal identifiable information contained in their e-mail address. Guidelines for an ethical conduct in performing crowdsourcing research should be consulted^47^.

The crowdsourced population in this study was substantially larger and presumably more diverse^18^ than in our in-lab investigation. Clearly crowdsourcing experiments were less controlled due to the lack of personal interaction with the investigators and observation of task execution as well as lack of control of correctness of demographic responses. In addition, subjects performed the task with their own equipment (computers and keyboards) and at a time and place of their choosing. With sample sizes above 100, motor learning data led to better mean estimations compared with the in-lab experiments (Supplementary Table 1) and collecting behavioral data using crowdsourcing was very time efficient (average data collection time was 16 h and 26 min per experiment). At sample sizes comparable with typical in-lab experiments, variability of crowdsourced behavioral motor data is higher, likely due to the more heterogeneous pool of participants^18^ and a less controlled setting. It is known, that sometimes large differences in sample composition among Amazon Turk workers are observed, even between studies with several thousand respondents^27^. As expected and consistent with previous reports^18^, a proportion of participants in our experiments did not follow instructions correctly (Table 1), possibly due to misunderstanding, inattention or negligence, and had to be excluded. This emphasizes the need to develop prospective well-defined plans for data quality control (see our prospective approach in “Methods”, “Data Analysis” section). Taken together, we experienced crowdsourcing as a very useful environment to study motor learning. Features that made our task amenable to study through crowdsourcing included the simple experimental design, accessible hardware, and short task duration as well as the clear definitions of correct adherence to instructions for post-hoc data quality check. However, experimental designs involving prolonged task durations or longitudinal measurements have also been successfully implemented^48,49^. Finally, particular care should be taken to properly randomize groups, thereby accounting for rapid changes in the worker pool composition.

In summary we report a crowdsourcing approach to characterize dynamics of early motor skill learning. With large sample sizes we studied the temporal microdynamics of learning and found that performance improvements occur during short periods of rest rather than during practice. We then showed that these micro-offline gains cannot be explained by latent motor slowing due to performance fatigue. The new motor memory experienced within-seconds stabilization immediately after the end of each practice period. Altogether results reveal mechanisms behind offline learning at the microscale of seconds and are consistent with a rapid form of consolidation occurring at a much faster time-scale than previously acknowledged.

## Methods

### Participants

Participants were recruited from the Amazon Mechanical Turk Platform (MTurk). Qualifications for registered MTurk workers to view and work on the tasks were: >95% approval rate on all previous MTurk assignments, location in the United States, right-handedness and no previous participation in any sequence learning task offered by our lab. The project was approved by the Combined Neuroscience Institutional Review Board of the National Institutes of Health (NIH). All participants declared via button press that they agree to participate and acknowledge the outline and purpose of the study, the NIH-based research-nature of the task, their voluntary participation, the time commitment and payment, their mandatory age above 18 years old, information on data safety and after being given contact information. Sample sizes for each experiment were estimated based on prior studies of motor sequence learning on MTurk^20^. Sample sizes were 389 participants for Experiment 1 (224 female, mean ± s.e.m. age 39.6 ± 0.56), 373 for Experiment 2 (239 female, age 38.8 ± 0.65), 118 for Experiment 3 (67 female, age 35.4 ± 1.00), and 71 for Experiment 4 (37 female, age 35.63 ± 1.14). These sample sizes represent the total number of participants after exclusion of assignments demonstrating incomplete adherence to task instructions. For Experiment 2, which had 3 groups, tasks corresponding to each group were randomly assigned to MTurk workers in bundles of ten, i.e., the first 10 MTurk workers would be assigned to the same task. Participants were paid $2 (Experiments 1 crowdsourced group, 3, 4) or $1.5 (Experiment 2), equivalent to >$8 per hour. The time of the task being posted was midday on weekdays for each experiment. All tasks were posted at once within each experiment, except for Experiment 2 in which collection was done in two partitions for technical (monetary) reasons.

### Task

Participants learned a procedural motor skill task^5,22,50^. Instructions were given as descriptions and with pictures before the motor task began. The task was identical to previously reported studies^2^ except for the hardware used being participants’ own in their chosen environment. They used the nondominant, left hand to perform a sequence of five keypresses (4-1-3-2-4) as quickly and accurately as possible in response to instructions displayed on a monitor. Keypresses were applied using the numeric keys of computer keyboards with the pinky finger corresponding to button # 1, the ring finger to # 2, middle finger to # 3, and index finger to # 4. The numeric sequence was displayed on the monitor continuously during practice periods. Feedback was provided in the form of a dot appearing immediately after each keypress regardless of correctness (Fig. 1a). Keypress timing (ms) was recorded for behavioral data analysis. Each trial consisted of alternating practice and rest periods^13,51^. In Experiment 1, each practice period lasted 10 s and was followed by a rest period of 10 s for a total of 36 trials. In Experiment 2, group “early interference”, each practice period consisted of 10 s practicing of a target sequence (4-1-3-2-4), immediately followed by 10 s practicing of an interfering sequence (2-3-1-4-2), followed by a rest period of 20 s; in group “no interference”, each practice period consisted of 10 s practicing of the target sequence (4-1-3-2-4), followed by a rest period of 10 s, followed by 10 s practicing of the interfering sequence (2-3-1-4-2), followed by a rest period of 10 s and in group “no interference”, each practice period of the target sequence (4-1-3-2-4) lasted 10 s and was followed by a rest period of 30 s. Each group trained over 12 trials (Table 1 and Figs. 2–4). To minimize distractions, participants of groups “early interference” and “no interference” were prompted to tap any key (1–4) once during rest period at random timepoints at least 11 s after the last and 7 s before the next trial. Experiments 3 and 4 were like Experiment 1, but with variable practice period durations. In Experiment 3, each practice period lasted 5 s and was followed by a rest period of 10 s for a total of 48 trials, matching a total task duration of 12 min. In Experiment 4, each practice period lasted 5, 6, 7, 8, 9, or 10 s, jittered across a total of 41 trials and was followed by a rest period of 10 s, matching a total task duration of 12 min. In all experiments, participants were instructed to focus on the visually presented five-item sequence (during practice periods) or on the text “rest” (during rest periods) displayed on the monitor. After the task, participants were asked which hand they used to type the sequence, their age, and some basic demographic questions for institutional reporting requirements. Stimuli were programmed, presented and responses recorded with Psytoolkit^52,53^.

### Data analysis

#### Performance

Analysis of motor performance was done identically as previously reported^2^. Tapping speed was quantified as the average of the time intervals (in ms) between adjacent keypresses within correct sequences^24^ divided by 1000 (keypresses/s). Performance within each trial was calculated as the mean tapping speed of all correctly performed sequences (including correct sequences the participant has not completed by the end of the trial^5,54^). Number of correct sequences was calculated per trial, also including correct sequences the participant has not completed by the end of the trial.

Each assignment was checked for correct implementation and adherence to task instructions, which may be insufficient by some participants due to the less controlled experimental setting offered via crowdsourcing marketplaces. Incorrect implementation of instructions or adherence was defined by (i) completion of the task with the right hand (documented by given answer to question after the task), (ii) completion of only one sequence repetition beyond trial 1, (iii) keypresses consistently different from instructed sequence, (iv) response times following the prompt to tap any number during breaks in Experiments 3 and 4 indicative of lack of attention to the screen, (v) deterioration of tapping performance over consecutive trials after initial increase (>20% loss in speed), indicative of diminished effort, (vi) no registered responses in any trial.

Total session learning over all trials (online learning) was calculated as the difference between the mean tapping speed, or number of correct sequences, of the last and the first trial. Data were analyzed using custom written code on MATLAB 2017b.

#### Modeling of performance curves

Performance curves of mean tapping speed and number of correct sequences per trial, *B*(*t*), were fitted using an exponential function *L*(*t*):

1 $$B\left( t \right)\,\sim \,L(t) = k_1 + k_2 \cdot \left( {1 - e^{ - k_3t}} \right),$$

where *k*_1−3_ represent the initial performance (function intercept), maximum performance (function plateau) and learning rate (function slope) respectively; and *t* ∈ [1, +∞) represents trial. Parameters *k*_1−3_ were estimated by gradient descent, with the objective function defined as the root mean square error between *B* and *L* functions:

2 $$\mathop {{\min }}\limits_{{\boldsymbol{k}} \in {\Bbb R}^3} \frac{1}{T}\mathop {\sum }\limits_t^T \left( {B\left( t \right) - L(t)} \right)^2 + \lambda \left\| {{\boldsymbol{k}}_2^2} \right\|,$$

where $\lambda \left\| {{\boldsymbol{k}}_2^2} \right\|$ is the L2 regularization term with $\lambda = 0.1$. Lower and upper bound constraints for each parameter *k*_1−3_ were set to [−2, 0, 0] and [4, 9, 6], empirically derived by minimizing the mean squared error between modeled and real values over a range of parameters. Additional inequality constraints were set to *a* + 0.15*b* > 0 and *a* + *b* < 10, which control for a positive learning when one trial has been executed and a physiologically plausible range of performance increase, respectively. The parameters were derived from a grid search of parameters on a subset of learning curves. Both the boundaries and inequalities remained constant throughout all following experiments to avoid overfitting within each experimental condition.

#### Microscale learning

We studied trial-by-trial early learning by dissecting performance improvements occurring during practice (micro-online) and during rest (micro-offline) periods, as done previously^2^. Micro-online learning was defined as the difference in tapping speed between the first and the last correct sequence of a practice period. Micro-offline learning was the difference in tapping speed of the last correct sequence of a practice period and the first correct sequence of the next practice period (Fig. 1b, e). The tapping speed of incomplete sequences was averaged with the previous complete sequence. In the case of only one correctly performed sequence, the speed of that sequence served as the first and last tapping speed of each trial. To derive the micro-online and micro-offline contribution to early learning we calculated the sum over all early learning trials at the participant level. Early learning was defined as trial 1–5, based on significant performance decrements, starting by trial 6 in Experiment 1 (trial-by-trial two-tailed one-sample nonparametric permutation test, *P* < 0.05, uncorrected), which may spuriously inflate micro-offline learning values due to preceding motor slowing. Thus, five values (practice periods) were summed for micro-online, and four values (rest periods) were summed for micro-offline learning. Early learning was derived as the sum of all micro-online and micro-offline values. Given the requirement of at least two completed sequences per trial for the analysis of microscale learning dynamics, participants with a lower performance were excluded from analysis of Experiment 3 and 4. For better comparability of these experimental groups to the performance level of the original group recorded in Experiment 1, a corresponding performance threshold was applied to the group 1 experimental group in analysis of Experiment 3 and 4.

### Visualization

Visualization of performance data was done identically as previously reported^2^. Performance curve: the within-trials time-resolved representation of tapping speed for illustration of the performance curve in Fig. 1b, f, 2b, 3b, 4b was derived as follows: for each participant, the tapping speed at each of the 10,000 ms constituting one practice period was defined as the average inter-tap interval of the sequence the participant was executing at that moment. The duration of the execution of each sequence was defined as the time between the first keypress of that sequence (or the beginning of the practice period) and the first keypress of the next (or the end of the practice period). The participants’ time series were averaged at each millisecond to give the performance curve.

### Quantification and statistical analysis

Early learning including micro-online, micro-offline, and total early learning, as well as online learning over all trials were tested for significance using two-tailed nonparametric permutation tests, one-sampled (within group comparison) or two-sampled (across groups comparison). *P* values are reported uncorrected for each test. Statistically not different across group comparisons were tested for equivalence using TOST for equivalence^55^, tests with an equivalence interval of −0.05 to 0.05 and alpha level of 0.05. This tests if any group difference has at least a minimal effect size (0.05)^29^.

## Supplementary information

Supplementary Material

Reporting Sum

## Data Availability

Behavioral data are available upon request by contacting the Lead Contact, Marlene Bönstrup (marlene.boenstrup@googlemail.com).

## Code availability

Custom written code is available upon request by contacting the Lead Contact, Marlene Bönstrup (marlene.boenstrup@googlemail.com).

## References

[1] Dayan E, Cohen LG (2011). Neuroplasticity subserving motor skill learning. Neuron.

[2] Bonstrup M (2019). A rapid form of offline consolidation in skill learning. Curr. Biol..

[3] Squire LR, Genzel L, Wixted JT, Morris RG (2015). Memory consolidation. Cold Spring Harb. Perspect. Biol..

[4] Robertson EM, Pascual-Leone A, Miall RC (2004). Current concepts in procedural consolidation. Nat. Rev. Neurosci..

[5] Walker MP, Brakefield T, Hobson JA, Stickgold R (2003). Dissociable stages of human memory consolidation and reconsolidation. Nature.

[6] Muellbacher W (2002). Early consolidation in human primary motor cortex. Nature.

[7] Brashers-Krug T, Shadmehr R, Bizzi E (1996). Consolidation in human motor memory. Nature.

[8] Robertson EM (2019). Skill memory: mind the ever-decreasing gap for offline processing. Curr. Biol..

[9] Benwell NM (2006). Short-interval cortical inhibition and corticomotor excitability with fatiguing hand exercise: a central adaptation to fatigue?. Exp. Brain Res.

[10] Sharples SA, Gould JA, Vandenberk MS, Kalmar JM (2016). Cortical mechanisms of central fatigue and sense of effort. PLoS One.

[11] Kuhn YA, Keller M, Ruffieux J, Taube W (2017). Adopting an external focus of attention alters intracortical inhibition within the primary motor cortex. Acta Physiol..

[12] Baechinger M (2019). Motor fatigability as evoked by repetitive movements results from a gradual breakdown of surround inhibition. bioRxiv.

[13] Rickard TC, Cai DJ, Rieth CA, Jones J, Ard MC (2008). Sleep does not enhance motor sequence learning. J. Exp. Psychol. Learn Mem. Cogn..

[14] Pan SC, Rickard TC (2015). Sleep and motor learning: is there room for consolidation?. Psychol. Bull..

[15] Brawn TP, Fenn KM, Nusbaum HC, Margoliash D (2010). Consolidating the effects of waking and sleep on motor-sequence learning. J. Neurosci..

[16] Button KS (2013). Power failure: why small sample size undermines the reliability of neuroscience. Nat. Rev. Neurosci..

[17] Fraley RC, Vazire S (2014). The N-pact factor: evaluating the quality of empirical journals with respect to sample size and statistical power. PLoS One.

[18] Stewart N, Chandler J, Paolacci G (2017). Crowdsourcing samples in cognitive science. Trends Cogn. Sci..

[19] Camerer CF (2018). Evaluating the replicability of social science experiments in nature and science between 2010 and 2015. Nat. Hum. Behav..

[20] Kahn AE, Karuza EA, Vettel JM, Bassett DS (2018). Network constraints on learnability of probabilistic motor sequences. Nat. Hum. Behav..

[21] Censor N, Sagi D, Cohen LG (2012). Common mechanisms of human perceptual and motor learning. Nat. Rev. Neurosci..

[22] Censor N, Horovitz SG, Cohen LG (2014). Interference with existing memories alters offline intrinsic functional brain connectivity. Neuron.

[23] Walker MP, Brakefield T, Morgan A, Hobson JA, Stickgold R (2002). Practice with sleep makes perfect: sleep-dependent motor skill learning. Neuron.

[24] Vahdat, S., Fogel, S., Benali, H. & Doyon, J. Network-wide reorganization of procedural memory during NREM sleep revealed by fMRI. *Elife* **6**10.7554/eLife.24987 (2017). 10.7554/eLife.24987 PMC5593513 28892464

[25] Karni A (1995). Functional MRI evidence for adult motor cortex plasticity during motor skill learning. Nature.

[26] Fischer S, Nitschke MF, Melchert UH, Erdmann C, Born J (2005). Motor memory consolidation in sleep shapes more effective neuronal representations. J. Neurosci..

[27] Chandler J, Shapiro D (2016). Conducting clinical research using crowdsourced convenience samples. Annu Rev. Clin. Psychol..

[28] Shadmehr R, Brashers-Krug T (1997). Functional stages in the formation of human long-term motor memory. J. Neurosci..

[29] Cohen, J. Statistical power analysis for the social sciences. (Lawrence Erlbaum Associates, 1988).

[30] Krakauer JW, Shadmehr R (2006). Consolidation of motor memory. Trends Neurosci..

[31] Kukushkin NV, Carew TJ (2017). Memory takes time. Neuron.

[32] Cellini N, McDevitt EA (2015). The temporal dynamics of motor memory across wake and sleep. J. Neurosci..

[33] Mednick SC, Cai DJ, Shuman T, Anagnostaras S, Wixted JT (2011). An opportunistic theory of cellular and systems consolidation. Trends Neurosci..

[34] Ptak R, Schnider A, Fellrath J (2017). The dorsal frontoparietal network: a core system for emulated action. Trends Cogn. Sci..

[35] Maquet P (2000). Experience-dependent changes in cerebral activation during human REM sleep. Nat. Neurosci..

[36] Ramanathan DS, Gulati T, Ganguly K (2015). Sleep-dependent reactivation of ensembles in motor cortex promotes skill consolidation. PLoS Biol..

[37] Cohen N (2015). Peri-encoding predictors of memory encoding and consolidation. Neurosci. Biobehav Rev..

[38] Engel AK, Fries P (2010). Beta-band oscillations–signalling the status quo?. Curr. Opin. Neurobiol..

[39] Fioravante D, Regehr WG (2011). Short-term forms of presynaptic plasticity. Curr. Opin. Neurobiol..

[40] Squire LR, Alvarez P (1995). Retrograde amnesia and memory consolidation: a neurobiological perspective. Curr. Opin. Neurobiol..

[41] McGaugh JL (2000). Memory–a century of consolidation. Science.

[42] Goedert KM, Willingham DB (2002). Patterns of interference in sequence learning and prism adaptation inconsistent with the consolidation hypothesis. Learn Mem..

[43] Hardwick RM, Rottschy C, Miall RC, Eickhoff SB (2013). A quantitative meta-analysis and review of motor learning in the human brain. Neuroimage.

[44] Arias P (2015). Central fatigue induced by short-lasting finger tapping and isometric tasks: a study of silent periods evoked at spinal and supraspinal levels. Neuroscience.

[45] Rodrigues JP, Mastaglia FL, Thickbroom GW (2009). Rapid slowing of maximal finger movement rate: fatigue of central motor control?. Exp. Brain Res.

[46] Amar-Halpert R, Laor-Maayany R, Nemni S, Rosenblatt JD, Censor N (2017). Memory reactivation improves visual perception. Nat. Neurosci..

[47] APA. https://www.apa.org/science/about/psa/2016/06/changing-minds (2016).

[48] Daly TM, Nataraajan R (2015). Swapping bricks for clicks: crowdsourcing longitudinal data on Amazon Turk. J. Bus. Res..

[49] Kar, D., Fang, F., Delle Fave, F., Sintov, N. & Tambe, M. "'A Game of Thrones': When Human Behavior Models Compete in Repeated Stackelberg Security Games", Proceedings of the 14th International Conference on Autonomous Agents and Multiagent Systems (AAMAS 2015).

[50] Censor N, Dimyan MA, Cohen LG (2010). Modification of existing human motor memories is enabled by primary cortical processing during memory reactivation. Curr. Biol..

[51] Censor N, Dayan E, Cohen LG (2014). Cortico-subcortical neuronal circuitry associated with reconsolidation of human procedural memories. Cortex.

[52] Stoet G (2017). PsyToolkit: a novel web-based method for running online questionnaires and reaction-time experiments. Teach. Psychol..

[53] Stoet G (2010). PsyToolkit: a software package for programming psychological experiments using Linux. Behav. Res. Methods.

[54] Hardwicke TE, Taqi M, Shanks DR (2016). Postretrieval new learning does not reliably induce human memory updating via reconsolidation. Proc. Natl Acad. Sci. USA.

[55] Rogers JL, Howard KI, Vessey JT (1993). Using significance tests to evaluate equivalence between two experimental groups. Psychological Bull..

