# Supplementary material for: Mechanisms of offline motor learning at a microscale of seconds in large-scale crowdsourced data
Source: NPJ Sci Learn. 2020 Jun 4;5:7. doi: 10.1038/s41539-020-0066-9 (PMC7272649; doi:10.1038/s41539-020-0066-9)
Supplement: Supplementary file 1 — Supplementary Material [file 41539_2020_66_MOESM1_ESM.docx]

**SUPPLEMENTARY MATERIAL**

**TITLE**

Mechanisms of offline motor learning at a micro-scale of seconds in large-scale crowdsourced data

**AUTHORS**

Marlene Bönstrup^1^ *; Iñaki Iturrate^1^; Martin N. Hebart^2^; Nitzan Censor^3^; Leonardo G. Cohen^1^

**AFFILITATIONS**

^1^Human Cortical Physiology and Neurorehabilitation Section, National Institute of Neurological Disorders and Stroke, Bethesda, Maryland 20814, USA

^2^Laboratory of Brain and Cognition, National Institute of Mental Health, Bethesda, Maryland 20814, USA

^3^School of Psychological Sciences and Sagol School of Neuroscience, Tel Aviv University, Tel Aviv 69978, Israel

**CORRESPONDING AUTHORS**

Marlene Bönstrup, MD or Leonardo G Cohen, MD

Human Cortical Physiology and Neurorehabilitation Section

National Institute of Neurological Disorders and Stroke, NIH

Building 10, Room 7D50

Bethesda, MD 20892

Phone: 301-496-8511

Fax: 301-402-7010

Email: [marlene.boenstrup@googlemail.com](mailto:marlene.boenstrup@googlemail.com) or cohenlg@ninds.nih.gov


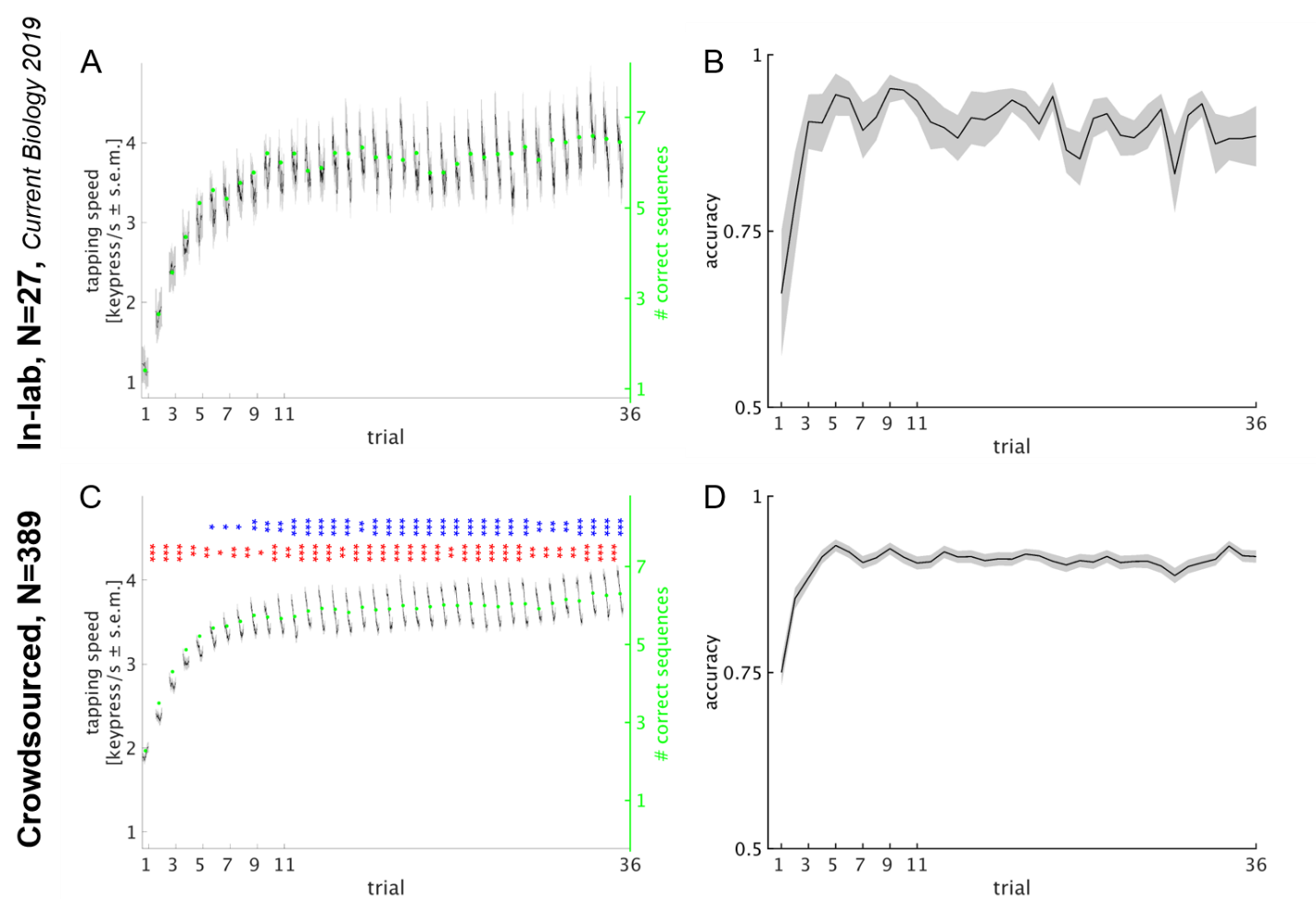


**Supplementary Figure 1 Experiment 1: Early learning of a new skill occurs largely offline. Replication in a large crowdsourced sample**

**A, B,** in-lab experiment, **C, D** crowdsourced experiment

**A, C:** Performance curves over all 36 trials. Skill was measured as the average inter-tap interval within correct sequences (tapping speed measured in keypresses/s, mean + s.e.m.) ^1^ and as the average number of correct sequences per trial (shown as green dots) ^2-5^. Note the similarity of in-lab and online-acquired learning curves. Stars in **C** indicate the significance level of trialwise micro-online (blue) and micro-offline (red) learning (two-tailed one-sample non-parametric permutation test, **P* < 0.05, ***P* < 0.01, ****P* < 0.001).

**B, D:** Accuracy was quantified as 1 minus the number of erroneous relative to correct keypresses in each trial ^6,7^. In the in-lab experimental group, accuracy increased rapidly over the first three trials and then stayed constant for the remaining 33 trials. In the crowdsourced experimental group, accuracy increased over 5 trials and then remained constant at a comparable level.


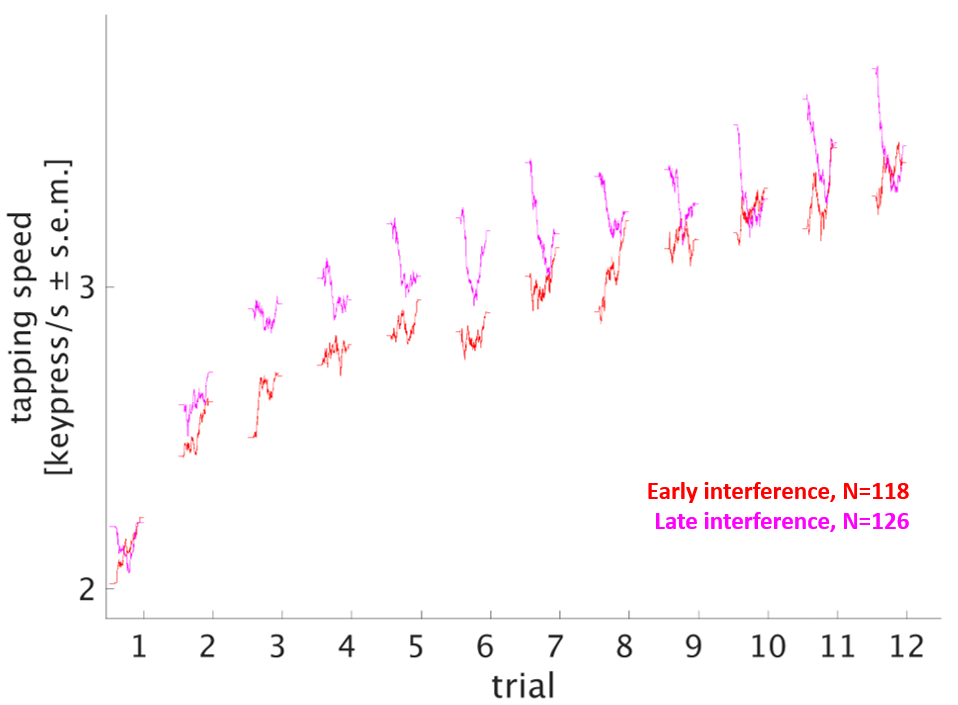


**Supplementary Figure 2 Experiment 2: Performance curve of interference sequences.** Learning of the target sequence 4-1-3-2-4 was interfered by learning of another sequence 2-3-1-4-2 either immediately (early interference, N=118, red), or 10s after (late interference, N=126, magenta) each practice period. Skill was measured as the average inter-tap interval within correct sequences (tapping speed measured in keypresses/s) ^1^ and as the number of correct sequences ^2-4,7^. The performance curve of the interference sequence of each group is displayed.


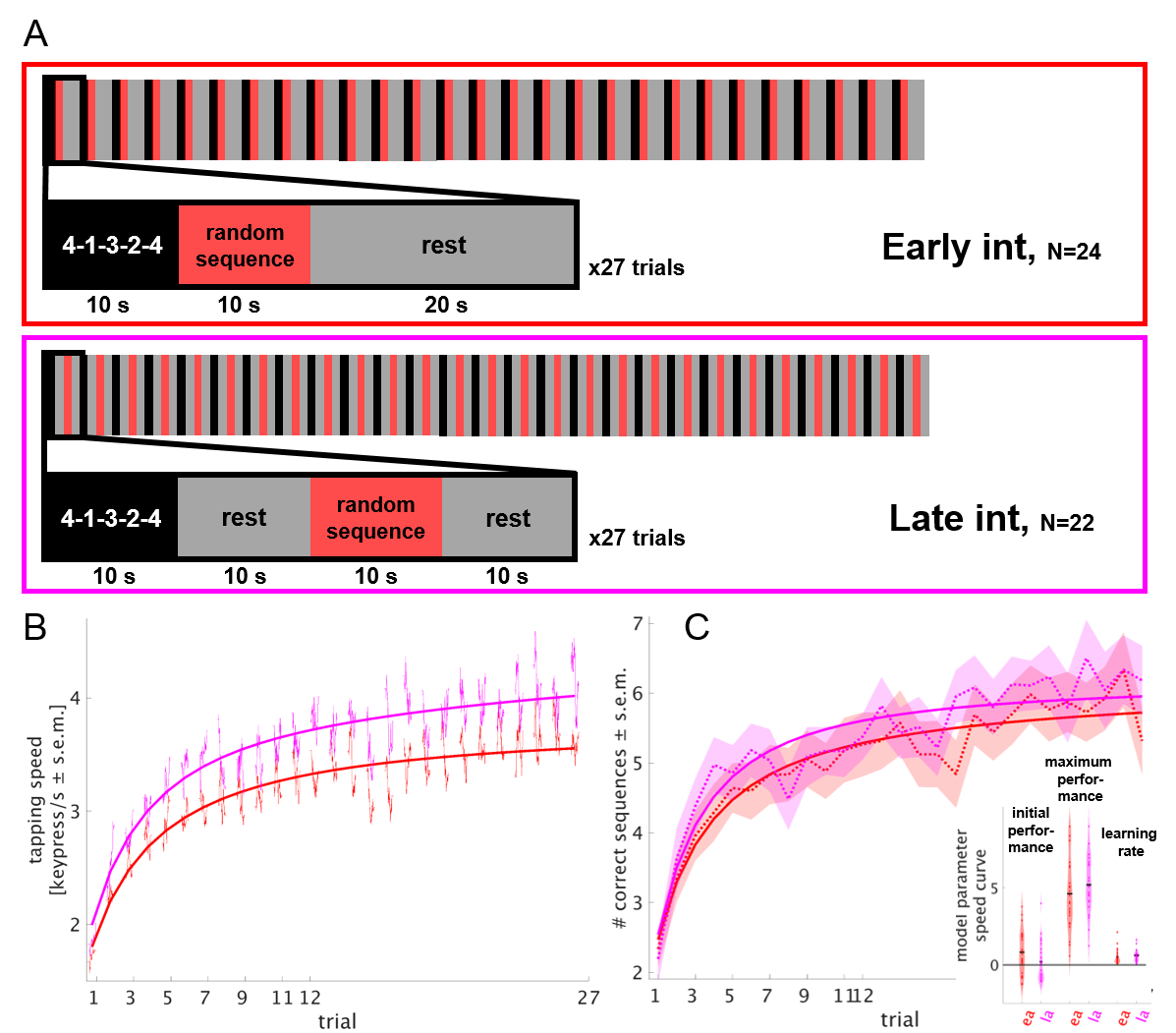


**Supplementary Figure 3 Stabilization of motor skill during short periods of rest in an in-lab collected experiment.**

Task: Learning of the target sequence 4-1-3-2-4 was interfered by learning of random other sequences (4-2-3-1-4; 2-3-1-4-2; 3-1-4-3-2; 1-4-3-4-2; 3-1-2-1-4; 3-2-4-2-1) either immediately (Early int, N=24, first row), or 10s after (Late int, N=22, second row) each practice period. To avoid proactive interference of late interference on the following trial, a rest period of 10s was introduced and the rest period in the early interference group matched to 20s. Training consisted of 27 trials (each inset shows a single trial). In each practice period (target or interfering sequence), participants were asked to repetitively tap the sequence indicated on the screen as fast and accurately as possible using their left, non-dominant hand. **B,** Skill was measured as the average inter-tap interval within correct sequences (tapping speed measured in keypresses/s) ^1^ and **C,** as the number of correct sequences ^2-4,7^. The performance curve of each group (red: early interference, magenta: late interference; mean + s.e.m.) is overlaid with the average of modelled performance curves. Note that the late interference group shows a steeper rise in early (trial 1-5) and higher level during later trials (6-27). Modelling of the number of correct sequences (trial 1-12 as in Experiment 2) revealed no significant group differences in the model parameters initial performance (*P* = 0.10), maximum performance (*P* = 0.28) and learning rate (*P* = 0.15, two-sample non-parametric permutation test).


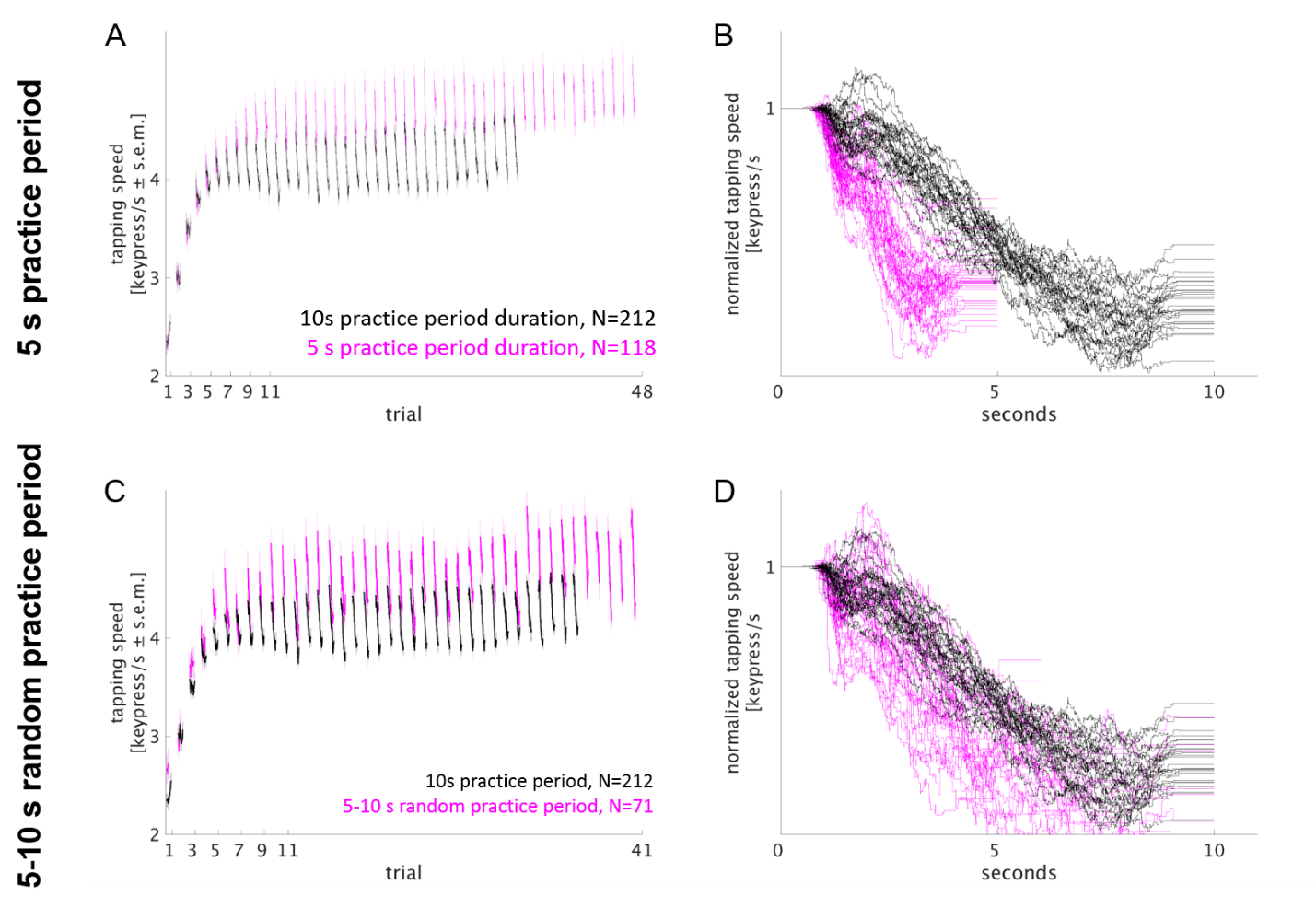


**Supplementary Figure 4 Experiment 3 and 4: Training under reduced or unexpected practice period duration shows comparable micro-offline gains.**

**A, C:** Performance curves over all trials of the 12 minutes training session for experiment 3 (**A**, 5 s practice period duration) and experiment 4 (**C**, unpredictable practice period duration (5, 6, 7, 8, 9 or 10 s practice period duration). Skill was measured as the average inter-tap interval within correct sequences (tapping speed measured in keypresses/s, mean + s.e.m.) ^1^ and is given for each experimental group (magenta) and the reference group with 10s practice period duration (black). In both experimental groups, performance ceiling was higher compared to the reference group.

**B, D:** Stacked performance curves of late learning trials (11-48 or 41). Performance is averaged within participants for each trial and normalized to a speed of 1 keypress/s of the first sequence. In the 5s practice period duration experimental group (experiment 3, **B**, magenta), performance decrements started earlier and were less pronounced than in the 10s practice period duration reference group (black). In the unpredictable practice period duration experimental group (experiment 4, **D**, magenta), performance decrements started earlier or at the same time and were equally pronounced as in the 10s practice period duration reference group (black).

**Supplementary Table 1. Estimators of the Mean.** For each experiment, variance and standard error (variance normalized by sample size) are given. Crowd-soured sample sizes above 100 yield lower variance.

| **Group** | **Variance**  **(keypresses/s ^2^)** | **Standard Error**  **(keypresses/s)** |
| --- | --- | --- |
| **Experiment 1, in-lab group**  **N=27** | 1.97 | 0.26 |
| **Experiment 1, crowdsourced group N=389** | 1.70 | 0.07 |
| **Experiment 2**  **N=373** | 1.62 | 0.07 |
| **Experiment 3**  **N=118** | 1.80 | 0.12 |
| **Experiment 4**  **N=71** | 2.09 | 0.17 |

**REFERENCES**

1 Vahdat, S., Fogel, S., Benali, H. & Doyon, J. Network-wide reorganization of procedural memory during NREM sleep revealed by fMRI. *Elife* **6**, doi:10.7554/eLife.24987 (2017).

2 Karni, A. *et al.* Functional MRI evidence for adult motor cortex plasticity during motor skill learning. *Nature* **377**, 155-158, doi:10.1038/377155a0 (1995).

3 Fischer, S., Nitschke, M. F., Melchert, U. H., Erdmann, C. & Born, J. Motor memory consolidation in sleep shapes more effective neuronal representations. *J Neurosci* **25**, 11248-11255, doi:10.1523/JNEUROSCI.1743-05.2005 (2005).

4 Censor, N., Horovitz, S. G. & Cohen, L. G. Interference with existing memories alters offline intrinsic functional brain connectivity. *Neuron* **81**, 69-76, doi:10.1016/j.neuron.2013.10.042 (2014).

5 Walker, M. P., Brakefield, T., Hobson, J. A. & Stickgold, R. Dissociable stages of human memory consolidation and reconsolidation. *Nature* **425**, 616-620, doi:10.1038/nature01930 (2003).

6 Hardwicke, T. E., Taqi, M. & Shanks, D. R. Postretrieval new learning does not reliably induce human memory updating via reconsolidation. *Proc Natl Acad Sci U S A* **113**, 5206-5211, doi:10.1073/pnas.1601440113 (2016).

7 Walker, M. P., Brakefield, T., Morgan, A., Hobson, J. A. & Stickgold, R. Practice with sleep makes perfect: sleep-dependent motor skill learning. *Neuron* **35**, 205-211 (2002).
